# Rate of de novo mutations in the three-spined stickleback

**DOI:** 10.1038/s41437-025-00767-9

**Published:** 2025-06-12

**Authors:** Chaowei Zhang, Kerry Reid, Mikkel Heide Schierup, Hongbo Wang, Ulrika Candolin, Juha Merilä

**Affiliations:** 1 Area of Ecology & Biodiversity, School of Biological Sciences, The University of Hong Kong, Hong Kong, Hong Kong SAR China; 2 Bioinformatics Research Centre, Aarhus University, Aarhus, Denmark; 3 Research Program in Organismal & Evolutionary Biology, Faculty of Biological and Environmental Sciences, University of Helsinki, Helsinki, Finland

**Keywords:** Mutation, Evolutionary genetics

## Abstract

As a fundamentally important genetic parameter and evolutionary force, germline mutation rates have many applications in evolutionary biology. However, accurate estimates of de novo mutation (DNM) rates are still relatively scarce, even for extensively studied evolutionary biology models. We estimated DNM rates for the three-spined stickleback (*Gasterosteus aculeatus*), the ‘supermodel’ of ecology and evolutionary biology. Using a large number of family trios sequenced to 45x coverage, we identified 115 unique mutations genome-wide and estimated the DNM rate at *µ* = 5.11 × 10^−9^/bp/gen without any detectable sex bias. The localised DNM rate was found to be positively correlated with the recombination rate, supporting the notion that recombination is a mutagenic process. Correlations between *µ* and genomic characteristics of studied species and the related nine-spined stickleback (*Pungitius pungitius*) revealed a high degree of similarity, suggesting that despite 17.5 million years of independent evolution, the mutational processes in the two species appear to have been conserved.

## Introduction

De novo mutations (DNMs) are the ultimate source of new genetic variation, and the rate, *µ*, is a central parameter for population and evolutionary genetic inference. However, until recently, direct estimates of *µ* were hard to obtain, but the rapid development and refinement of modern sequencing technologies and computational tools have led to an increase in *µ* estimates from various non-model species (Smeds et al. 2016; Koch et al. 2019; Wang, et al. 2022; Sendell-Price et al. 2023; Wang and Obbard 2023; Zhang et al. 2023). Yet, there are still strong taxonomic biases in the available estimates with mammalian, and those of primates in particular, being overrepresented, whereas those of birds, reptiles, amphibians and fish are still rare among vertebrates (Wang and Obbard 2023; but see: Bergeron et al. 2023). Furthermore, there is a clear bias towards estimates from well-established model species (e.g. *H. sapiens, M. musculus, D. melanogaster,C. elegans, Z. mays, A. thaliana*) being based on a much larger number of mutations than those from non-model species. Consequently, most direct estimates of *µ* for the non-model species are based on only a few parent-offspring trios and therefore have broad confidence intervals covering several fold ranges of *µ*-values (e.g. Wang and Obbard 2023 their Fig. 2; Bergeron et al. 2023 their Fig. 1a). Uncertainty around these estimates is further compounded by difficulties controlling false positive and negative rates when estimating *µ* from parent-offspring data (Yoder and Tiley 2021; Bergeron et al. 2022). Hence, there is a definitive need to obtain more accurate estimates of *µ* from many non-model species based on larger numbers of trios.

**Fig. 1:**
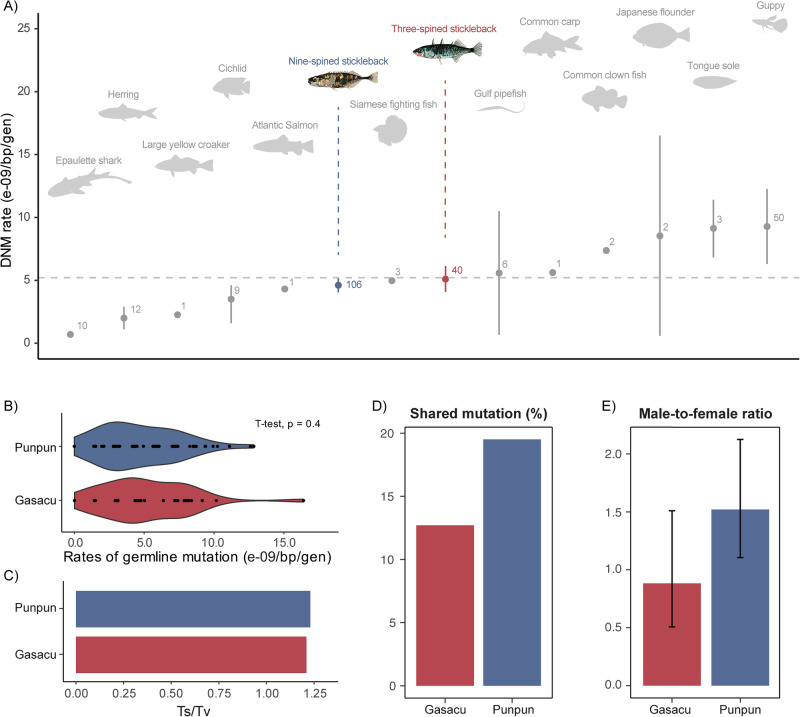
Germline mutation rates (*µ*) and their characteristics in three- (Gasacu) and nine-spined sticklebacks (Punpun). **A** Comparison of *µ* in sticklebacks with those in other fishes (Statistics next to the data points show the number of trios included for each species, with references listed in Supplementary Data), **B** a violin plot of per-trio de novo mutation (DNM) rates, **C** the transition (Ts) to transversion (Tv) ratio, **D** the percentage of shared mutations among full-sibs, and **E** the parental origin (male-to-female ratio) of the DNMs in three- (red) and nine-spined (blue) sticklebacks.

The three-spined stickleback (*Gasterosteus aculeatus*) is a popular model system for ecological (Bell and Foster 1994), evolutionary (Colosimo et al. 2005; Lescak et al. 2015; Wootton 2009), behavioural (Von Hippel 2010; Bell et al. 2016, 2018; Norton and Gutiérrez 2019), physiological (Von Hippel et al. 2016) and genomic (Peichel and Marques 2017; Reid et al. 2021) research and it has been dubbed the “supermodel of ecology and evolutionary biology“ (Gibson 2005; Barber 2010). Despite the voluminous body of research tracking its evolutionary history in different parts of this distribution range (Orti et al. 1994; Mäkinen et al. 2006; Mäkinen and Merilä 2008; Shikano et al. 2010; Lescak et al. 2015; Fang et al. 2020a, b, 2021; Feng et al. 2022) and research devoted to deciphering genomic underpinnings of its adaptation to different environments (Jones et al. 2012; Fang et al. 2021; Roberts Kingman et al. 2021), no study has yet estimated its germline de novo mutation rates. Studies which have needed to parametrise three-spined stickleback mutation rate have used either substitution rates or generic approximations of *µ* (Liu et al. 2018; Ravinet et al. 2018; Varadharjan et al. 2019; Yamasaki et al. 2020; Dahms et al. 2022; Feng et al. 2022). Although DNM rate estimates are available for the sister species, the nine-spined stickleback (Zhang et al. 2023), the rate in three-spined sticklebacks can be different due to their independent evolutionary paths over the past 17.5 million years (Zhang et al. 2023).

The aim of this study was to estimate the rate of germline de novo mutations in the *G. aculeatus* and characterise its mutation spectrum using a large number of family trios sequenced to high coverage. To gain insights into the evolutionary conservation of mutational processes, we compared the obtained estimates with those of its relative, the nine-spined stickleback (*Pungitius pungitius*), previously obtained and analysed in a comparable manner.

## Material and methods

### Sampling and breeding

The fish used in this study originated from the Baltic Sea site Tvärminne, Finland (59°50’20”N; 23°12’15”E). The parental fish were caught with a beach-seine in May 2023 and brought alive to the Tvärminne Zoological Station’s aquaculture facilities. Five randomly selected males in breeding condition, as determined by their nuptial coloration, were placed into individual 80 L flow-through aquaria with nesting materials to enable nest building (Candolin 1997). When a male had built a nest, a gravid female was placed into the aquarium and allowed to spawn in the nest. Two females did not spawn within 2 h, and they were replaced by another two gravid females. After spawning, the female was removed, and the male was left to care for the eggs in the nest (about 80 eggs per pedigree). When the eggs started to hatch, and the first fry appeared outside the nest, the male was removed to prevent him from cannibalising the fry. The fry were fed *Artemia salina* larvae and allowed to grow until 1 week old. All individuals of a family (male, female and offspring) were preserved in 91% ethanol and stored at −20 °C before being preserved in 95% alcohol and shipped to The Merilä lab in Hong Kong for DNA extractions.

### DNA extraction and sequencing

Ten offspring were randomly picked from each pedigree, and the DNA was extracted from the fin clips of all samples using the DNEasy Blood and Tissue kit (Qiagen, Germany) with RNAse treatment following the manufacturer’s specifications. After evaluating the DNA purity and the concentration, the DNA samples were sent for PCR-free library preparation and whole-genome resequencing to a target depth of 45x coverage (paired-end 150 bp read length) using the DNBseq platform (Berry Genomics, China).

### Read mapping, variant calling and pedigree examination

The paired-end raw reads were mapped to the reference genome of three-spined stickleback (version 5, https://stickleback.genetics.uga.edu/downloadData/, an updated version to Peichel et al. 2020), using BWA-MEM (v0.7.17, Li and Durbin 2009). The paired reads were sorted, and the mate information was filled in by applying the ‘sort’ and ‘fixmate’ functions in SAMtools. (v1.16.1, Li et al. 2009). The duplicate reads were marked through PicardTools (v2.27.5; http://picard.sourceforge.net). The single nucleotide variants were called for each sample separately from these sorted BAM files using GATK (v4.3.0.0; Van der Auwera and O’Connor 2020) HaplotypeCaller in ERC mode, following the best practices workflow of GATK (Poplin et al. 2017). The resulting gVCF files were then combined and jointly genotyped using GATK CombineGVCFs and GenotypeGVCFs modules for all samples involved. Utilising the hard-filtered output sets of SNPs and indels from this step, Base-quality score recalibration (BQSR) was carried out to the sorted BAM files in GATK, after which the variants were called again from the final BAM files for each individual, and the generated gVCF files were combined in the same manner as mentioned for each parent-progeny trio.

Before the further steps, the parent-offspring relationships within each pedigree were confirmed by estimating the probabilities of identity-by-descent (IBD) with PLINK (v1.90; Chang et al. 2015). The ratio of probabilities of sharing 0, 1 and 2 haplotypes (Z_0_:Z_1_:Z_2_) between a parent and its offspring was expected to be close to 0:1:0. We identified a mislabeled mother and removed one pedigree from our dataset by this method.

### Identifying putative de novo mutations

For each parent-offspring trio, only SNPs violating Mendelian expectations were considered candidate point mutations. In other words, one of the two alleles of the offspring should be absent from its parents to count as a mutation. Given the rarity of alternative alleles mutating back to reference alleles, we retained only those variants where the parents were homozygous for the reference allele (0/0) and the offspring were heterozygous (0/1). The site filters (also known as the hard filtering suggested by GATK best pipeline, Poplin et al. 2017) and the individual filters were applied following the “Mutationathon” guidelines (Bergeron et al. 2022), as detailed in Supplementary Table 1 and Zhang et al. (2023).

To further control the false positive rates, the putative DNMs were validated by loading the final alignments of each trio in the visualisation tool, IGV (Thorvaldsdóttir et al. 2013), and only sites where the genotypes were consistently supported by the aligned reads were retained. Candidate DNMs were excluded based on the following criteria: first, if more than one read was detected in the parents which carried the same variant as their offspring; and second, if the offspring was incorrectly identified as a heterozygote due to poor mapping. A screenshot of a DNM candidate that passed the IGV filter as a reference can be found from the Supplementary Fig. 6. To avoid subjective judgement, the number of reads at the DNM candidates was double-checked in ‘bam-readcount’ (Khanna et al. 2022) to exclude mismatches between the final BAM files and the realigned VCF files. We also invited two additional readers to review the IGV screenshots of the DNM candidates, and only those congruently validated by all readers were retained.

### De novo mutation rate estimation

The per-generation point mutation rate was estimated for each sample by:

1 $$\mu =\frac{{n}_{{\rm{DNMcandidate}}}\times (1-{\rm{FDR}})}{2\times {\rm{CS}}\times (1-{\rm{FNR}})}$$

And for all samples by a zero-inflated method:

2 $$\mu =\frac{sum({n}_{{D}{N}{M}\,{a}{f}{t}{e}{r}\,{m}{a}{n}{u}{a}{l}\,{c}{u}{r}{a}{t}{i}{o}{n}})}{2\times sum{(}{C}{S}{)}\times (1-mean{(}{F}{N}{R}{)})}$$

The callable genome size (CS) was the number of sites within the filtered final bam file for each offspring that passed the depth filtering within its trio family (0.5DP_trio_ < DP_child_ < 2DP_trio_), and where the both parents are homozygotes. The false discovery rate (FDR) was the proportion of DNM candidates removed by the IGV and the bamcount inspections mentioned in the above section:

3 $${\rm{FDR}}=\frac{{n}_{{\rm{false}}\,{\rm{positives}}\,{\rm{identified}}\,{\rm{in}}\,{\rm{IGVtools}}}}{{n}_{{\rm{all}}\,{\rm{candidates}}\,{\rm{after}}\,{\rm{individual}}\,{\rm{filters}}}}$$

The false negative rate (FNR) was inferred from the percentage of true offspring heterozygotes, when the genotypes of the parental sites were one 0/0 and one 1/1, that had been deleted by the allelic balance (AB) filtering (AB < 0.3 and AB > 0.7):

4 $${\rm{FNR}}=\frac{{n}_{{\rm{true}}\,{\rm{heterozygotes}}\,{\rm{being}}\,{\rm{removed}}\,{\rm{by}}\,{\rm{AB}}}}{{n}_{{\rm{true}}\,{\rm{heterozygotes}}}}$$

### Genome-wide mutation rate and its comparison with recombination rate

We calculated the genome-wide mutation and recombination rate for the entire dataset using a 5 Mb sliding window and a 1 Mb step length. Applying the linkage map available for three-spined stickleback (Kivikoski et al. 2022), the recombination fractions were calculated with the following equation:

5 $$r(cM/Mb)=\frac{genetic\,distance}{physical\,distance}$$

Since recombination is known to be mutagenic, we tested for correlation between mutation and recombination rates. To align with the linkage map, the detected DNMs were lifted over to the corresponding version of the reference genome (Peichel et al. 2017) by utilising the provided chain file (https://stickleback.genetics.uga.edu/downloadData/). We then estimated the per-5Mb DNM count for the same sliding windows. Pearson product-moment correlation tests were conducted to see if the per-window size mutation rate correlates with the recombination rate.

### Mutation spectrum and genomic context

The CpG islands (Henceforth: CGIs), which are the regions of significantly higher levels of CpG dinucleotides compared to the genome as a whole, were predicted with the default settings using the “twoBitToFa” module and the cpg_lh script in the UCSC tools (http://genome.ucsc.edu/cgi-bin/hgTrackUi?g=cpgIslandExt, Miklem and Hillier 2022). The detected CGIs were classified into intergenic, intronic, exonic, transcription start site (TSS), and transcription termination site (TTS) CGIs, based on their genomic location. With the above statistics, the per-5Mb CpG densities and gene densities were quantified similarly to what was done for the genome-wide mutation/recombination frequencies.

The detected DNMs were classified using snpEff (v5.1f) and a manual verification according to the annotation file. To generate the mutation spectrum, these mutations were also stratified into six groups according to their mutation forms (A > C, A > T, A > G, C > A, C > G, C > T), with a special inspection on the C > T mutations on the CpG sites (the 7th group). Among these mutation types, A > G and C > T are the transitions (Ts), and the rest are transversions (Tv). A Chi-squared test was used to test whether there was any bias in strong base pairs (C:G) mutating to weak ones (A:T), or vice versa.

To examine if de novo mutations were biased to originate from males or females, all the identified DNMs were phased back to their parent of origins with a read-backed phasing method, POOHA (https://github.com/besenbacher/POOHA). The per-sample percentage of the paternal sites was then calculated to infer the paternal-to-maternal ratio (alpha) of the DNMs. Additionally, mutations occurring at the early stages of meiosis and shared among the full-sibs were also identified and counted.

### Comparison to nine-spined stickleback (*Pungitius pungitius*)

To compare the three-spined stickleback mutation rates and patterns with those of the nine-spined stickleback, we estimated the per-5Mb genome-wide mutation frequencies, recombination frequencies, CpG densities, and the gene densities for *P. pungitius* using the same methods described in the above sections using data from Zhang et al. (2023). The pairwise synteny between the two reference genomes (*G. aculeatus* V4 vs. *P. pungitius* V7) was visualised using MCscan (Tang et al. 2024). The recombination fraction was obtained from a previously available linkage map of the eastern European lineage of *P. pungitius* (Kivikoski et al. 2022). To make the annotations of *P. pungitius* comparable to the three-spined stickleback, we again lifted over the NCBI annotations for the version 6 reference genome (GCF_902500615.1) to the version of the published mutation data and the linkage map (version 7, GCA_902500615.3, Kivikoski et al. 2021) with removing the non-protein-coding genes by the pygtftk tool (Lopez et al. 2019). In total, more than 20 thousand genes were annotated across the autosomal genomes for both species, and the genomes were subsequently divided into exonic (including protein-coding regions and untranslated regions, UTRs), intronic, and intergenic regions. Mutations located within the repetitive regions were identified according to the NCBI annotations for the both species.

*t*-tests were conducted to assess the significance of the differences between the two species. To investigate the relationship between various factors and mutation rates, generalised mixed linear models (using a Poisson distribution) were constructed in lme4 (v1.1-36, Bates et al. 2015) using combined data from the two species. In these models, species nested with the pedigree was treated as a random effect, while other factors were treated as fixed effects in three separate models:

6 $${\rm{\#DNM}} \sim {\rm{Factors}}(\left.{\rm{Recombination\; rate}}\right|\left.{\rm{CpG\; content}}\right|{\rm{Gene\; density}})+\left(\left.1\right|{\rm{Species}}\right)$$

A path analysis was conducted to assess the direct and indirect impact of CpG content and recombination rates on DNM rates in R package “lavaan” (v0.6-19, Rosseel 2012), and to determine the pathway and direction they correlated with.

## Results

### De novo mutation rate in three-spined sticklebacks

Forty-eight samples belonging to four pedigrees of unrelated parents, each with 10 full-sib offspring, were whole-genome resequenced. The final bam files from the raw reads mapped to the reference genome had a mean coverage of depth of 45.4× before variant calling. Over 526 thousand sites were retained across the 40 trios after filtering for Mendelian violations in all autosomal variants (excluding those on the sex chromosomes Y and XIX), averaging 13,152 sites per progeny. After applying a series of filters, 115 unique de novo mutations (DNMs) were detected (see Supplementary Table 1 for more details). The average callable genome size was 87.40% of the entire autosomal genome (363.45 Mb), and the mean false negative rate (FNR) was 7.44%. The overall average per-generation point DNM rate was estimated to be *µ* = 5.11 × 10^−9^/bp/gen (95% confidence interval: 4.08–6.14 × 10^−9^/bp/gen, with zero-inflated method being 5.07 × 10^−9^/bp/gen), and it resided in the middle of all current available DNM rates in fish, with the second narrowest 95% CIs of all fish species (Fig. 1A). Assuming a generation time of 3 years for the marine three-spined sticklebacks, the yearly mutation rate would be, on average, 1.70 × 10^−9^/bp/year. The per-generation DNM rates did not differ between marine three- and nine-spined sticklebacks (Fig. 1B, *t*_64.43_ = 0.85; *p* = 0.40).

### Mutation characteristics and spectrum

**Fig. 2:**
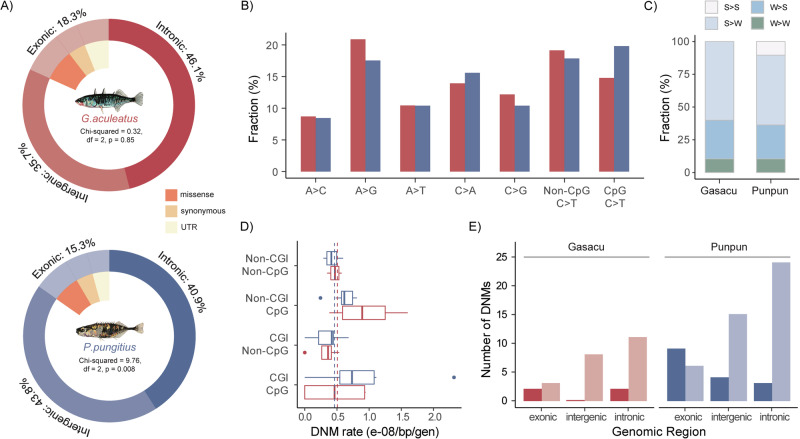
Genomic landscape of de novo mutations (DNMs) in three- (*G. aculeatus*) and nine-spined (*P. pungitius*) sticklebacks. **A** Distribution of DNMs on different genomic regions. **B** Comparison of mutation spectra between three- (red) and nine- (blue) spined sticklebacks. **C** DNM composition based on changes in pairing types. **D** Pedigree-level DNM rates in CpG or Non-CpG sites within or outside CpG islands (CGI). The two dashed lines depict genome-wide DNM rates for the two species. **E** Number of unique CpG DNMs located inside CGI (darker bars) or outside CGI (lighter bars) in the two species.

Out of all distinct DNMs identified, 54.8% were transitions (Ts) and 45.2% were transversions (Tv), resulting in a Ts:Tv ratio of 1.21, which was similar to that in the nine-spined stickleback (Fig. 1C). In both species, we observed about twice as many DNMs mutating from the strong (C:G) to weak (A:T) base pair (S > W) compared to the opposite direction (W > S), and very few DNMs mutating within its own pairing type (S > S or W > W, Fig. 2C). This finding deviates significantly from the null expectation of equal mutation rates, given the GC proportion after correction by the callable fractions (*G. aculeatus*: *χ*² = 11.94, df = 1, *p* = 5.51e−04; *P. pungitius*: *χ*² = 29.09, df = 1, *p* = 6.92e−08). This GC mutation bias was also evident in the mutation spectrum (Fig. 2B). In addition to the typically high proportion of C > T mutations, whether at CpG sites or not, an elevated number of C > A mutations was observed in both species as well (Fig. 2B).

In total, 21, 53 and 41 DNMs were found in the exonic, intronic, and intergenic regions in *G. aculeatus*, while these numbers were 47, 126 and 135 in *P. pungitius*, respectively. Adjusted for the callable fractions of the genome, the DNM locations did not deviate significantly from a random distribution across genomic features in *G. aculeatus* (*χ*² = 0.32, df = 2, *p* = 0.85), but slightly so in *P. pungitius* with fewer DNMs detected within the intronic regions than expected (*χ*² = 9.76, df = 2, *p* = 0.008, Fig. 2A). Among the exonic mutations for the two species, 45.6% were missense, 23.5% were synonymous DNMs and 30.9% occurred on untranslated regions (UTRs, Fig. 2A). These exonic mutations were all categorised as being either moderate or low impact by snpEff, and no loss-of-function mutations were observed in either dataset.

The total length of the autosomal CpG islands (CGIs) in three-spined sticklebacks was estimated to be 11.6% of the entire callable genome. 9.6% of the unique DNMs were located within these areas, with five DNMs being C > T among which three situated on CpG sites. There was a clear inflation in DNM rates in the non-CGI CpG sites in three-spined sticklebacks (Fig. 2D), although this was not significantly different from a null expectation given the GC contents of the CGI or non-CGI regions, controlled for the callable fractions (*χ*² = 0.13, df = 1, *p* = 0.72). More CGI CpG DNMs are located on exons than the other regions in both species (Fig. 2E), but this does not deviate significantly from the null hypothesis given the number of CGI-CpG sites located within each genomic feature (*χ*² = 1.12, df = 2, *p* = 0.57 for *G. aculeatus* and *χ*² = 1.94, df = 2, *p* = 0.38 for *P. pungitius*).

### Weak sex bias of DNM rates in sticklebacks

In total, 64.3% unique DNMs detected in the offspring were traced back to their parents-of-origin, among which, on average, 46.9% (95% CI: 33.6%–60.2%) DNMs originated from the fathers. This translated to an α value (male-to-female ratio) at 0.88 (95% CI: 0.51–1.51), which was not significantly different from that of the nine-spined stickleback (Fig. 1E, *t*_61.43_ = −1.75, *p* = 0.085). Unlike in *P. pungitius* in which a significantly higher proportion of CpG sites were inherited from the paternal than the maternal side (Supplementary Fig. 1), the proportions were equal in the three-spined stickleback (*χ*^2^ = 0.22, df = 1, *p* = 0.64).

Additionally, less DNMs were found to be shared among full-sibs in the three-spined than in the nine-spined sticklebacks (12.7% vs. 19.5%, Fig. 1D). Except for DNM shared between two offspring, sites shared between 3 and 4 siblings were observed on rare occasions. Due to the limited number of shared mutations being phased back to their parental origins (7 and 48 in three- and nine-spined sticklebacks, respectively), a combined dataset of the two species were analysed, and no sex bias was detected neither for the shared (Maternal:Paternal = 26:29, *χ*^2^ = 0.16, df = 1, *p* = 0.27) nor for the non-shared DNMs (Maternal:Paternal = 108:125, *χ*^2^ = 1.24, df = 1, *p* = 0.69).

Respectively, 16.6% and 17.9% of the callable genome were repetitive regions for three- and nine-spined sticklebacks, and 34.8% and 28.6% DNMs were located within these regions. Among these, nine and twenty mutations were shared by full-sibs, while the majority of DNM candidates were not shared within these regions (77.3% and 77.5% for both species). Mutation rates in repetitive regions were higher than in the rest of the genome, estimated at 9.03 and 6.77 × 10^−9^/bp/gen for three- and nine-spined sticklebacks, respectively.

### Mutation distributions and their correlation with the other genomic features

**Fig. 3:**
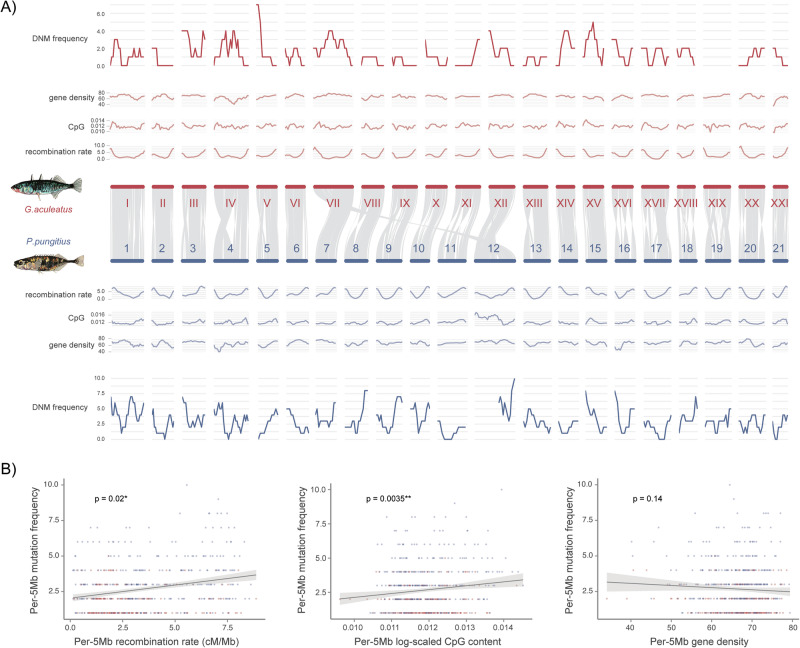
Genome-wide mutation rate and its correlation with the other genomic features. **A** The graphs, from the inner to the outer layers, displayed in order (1) the comparative synteny plot, the per-5Mb, (2) recombination rates, (3) CpG content, (3) gene density and (4) DNM frequencies. **B** Correlations between the genome-wide DNM frequencies within 5 Mb sliding windows and, from the left to right, (1) the recombination rates, (2) the CpG contents, and (3) the gene densities.

The synteny analyses demonstrated similar trends between the two species in their genome-wide features, including the mutation frequencies, recombination rates, the CpG dinucleotide contents and the gene densities (Fig. 3A). Notably, for the translocation identified between chr7 (1–13.2 Mb) in *G. aculeatus* and the pseudoautosomal region on LG12 (from 16.9 Mb) in *P. pungitius* (Fig. 3A), the de novo mutation rates were similarly high exceeding the genome-wide level, at respectively 9.95 × 10^−9^/bp/gen and 1.03 × 10^−8^/bp/gen. The localised recombination rates for these two translocated regions were also elevated to 4.01 and 4.44 cM/Mb, while the genome-wide values were 3.78 and 4.12 cM/Mb for the two species (Supplementary Fig. 3B).

Furthermore, these genomic features exhibited clear correlations with each other: DNM frequencies showed a significant positive correlation with CpG contents across 5 Mb sliding windows (*p* = 0.0035, Fig. 3B). Likewise, DNM frequencies were also a positive function of recombination rate (*p* = 0.020, Fig. 3A), but not that of gene density (*p* = 0.14, Fig. 3B). This was also evident in the inflated *µ* in CpG sites compared to the non-CpGs (Supplementary Fig. 5B), although we did not observe an obvious increase in *µ* on CGIs compared to those outside CGIs, possibly due to the rarity of mutations in sticklebacks (*t*_16.62_ = −0.75, *p* = 0.46; Supplementary Fig. 5A). Furthermore, a significant collinearity was observed between CpG contents and recombination rates (*p* = 5.74e−12, Supplementary Fig. 3A). The path analysis results indicated that CpG content indirectly impacted DNM rates by firstly affecting recombination rates (*p* < 2e−16) before impacting DNM rates (*p* = 0.001), while its direct impact on DNM rates was not significant (*p* = 0.13, Supplementary Fig. 4).

## Discussion

In spite of decades of focus on the three-spined stickleback as a model system for evolutionary genetic research, no estimate of de novo mutation (DNM) rate has emerged for this species until now. The estimated mutation rate *µ* = 5.11 × 10^−9^/bp/gen is very similar to that obtained for *P. pungitius* (Zhang et al. 2023), and also falls in the middle of the estimates available from other teleost fishes. Furthermore, the features of the mutation spectrum and genomic characteristics of DNMs in the three-spined stickleback are very similar to those of the nine-spined stickleback suggesting little divergence in mutational processes in spite of more than 17 million years of independent evolution.

DNMs in three-spined sticklebacks were distributed randomly across the genomic features (viz. exonic, intronic, or intergenic), while nine-spined sticklebacks show a slight enrichment of DNMs in intergenic regions. The latter result differs slightly from that reported in Zhang et al. (2023) because of the differences in annotation files utilised. As seen in humans (Panchin et al. 2016; Youk et al. 2020) and in nine-spined sticklebacks (Zhang et al. 2023), CpG sites outside CpG islands (CGIs) exhibit higher *µ* than the genome-wide average in three-spined sticklebacks, while CpG sites within CGIs have mutation rates similar to the overall *µ*. This is likely due to hypermethylation of the CpG outside CGIs and hypomethylation inside CGIs. This lower *µ* within CGIs likely contributes to the conservation and maintenance of CpG content across species. Additionally, CpG sites are known to be enriched near the transcription start sites (TSS) to regulate gene transcription (Jones 2012), and these CGIs are often hypermethylated (Youk et al. 2020). This could explain the inflated number of CpG DNMs in exons that was seen in both species in this study.

It is well-established that mutation rates are influenced by both intrinsic and extrinsic factors. For instance, there is evidence to suggest that recombination is a mutagenic process leading to errors during DNA replication causing double-strand breaks (DSBs) (Resnick 1976; Lercher and Hurst 2002; Halldorsson et al. 2019; Hinch et al. 2023; Palsson et al. 2025). We found a positive correlation between the localised mutation rate and recombination rate providing evidence for the mutagenic nature of the recombination process, and this is presumably more pronounced in the pseudoautosomal regions where both the mutation and recombination rates are elevated. Highly recombining genomic regions are also enriched for CpG mutations which exhibit increased mutation rates through deamination when methylated (Lindahl 1993; Zhou et al. 2020).

Sticklebacks lack the functional domain of *prdm9* (Shanfelter et al. 2019; Cavassim et al. 2022), which is known to modulate the occurrence of genome-wide recombination hotspots (Baudat et al. 2010; Myers et al. 2010; Parvanov et al. 2010). As a result, recombination is more likely to occur at the promoter-like regions (Singhal et al. 2015), including CGIs, causing the DNM rates to vary with the CpG content. Due to the stability in the distribution of GC content, the observed recombination and mutation hotspots are likely conserved for species lacking functional *prdm9* (Auton et al. 2013; Singhal et al. 2015). This could possibly explain why the three- and nine-spined sticklebacks exhibit similar trends in recombination rate and *µ* despite their long independent evolution. A recent study also demonstrated a similar correlation between CpG content and *µ* in dogs that have lost the entire *prdm9* (Zhang et al. 2024). The path analysis indicated that the estimated recombination rate impacted mutation rates, while being moderated by CpG content. However, given the low mutation rates in fish and the limited occurrence of DNMs within CGIs in our dataset, a more comprehensive analysis with larger sample size is needed to better understand the causality of these relationships.

The mutation spectrum observed in sticklebacks is generally similar to that of other vertebrates, with a higher prevalence of C > T and A > G substitutions compared to the other substitution types (Bergeron et al. 2023). Regardless of the presence of *prdm9*, GC-biased gene conversion (gBGC) has been found in various organisms originating from a repair bias of crossovers favouring G:C (S) over A:T (W) alleles (Birdsell 2002; Duret and Galtier 2009; Rousselle et al. 2019). This process promotes the segregation and fixation of W > S mutations with minor fitness effects, ultimately contributing to the increase in mutation load (Glémin 2010). However, as seen in other organisms, S > W DNMs in sticklebacks are also overrepresented compared to W > S ones (Kong et al. 2012; Wong et al. 2016; Wu et al. 2020; Campbell et al. 2021; Bergeon et al. 2023). The GC content at equilibrium will thus be a balance between the S > W bias of DNMs and the W > S bias of gene conversion. Despite these similarities, we observed a higher frequency of C > A mutations in sticklebacks compared to mammals, a trend also seen in other fish species (Bergeron et al. 2023). Given that mutation spectra are generally more similar among closely related species (Beichman et al. 2023), this phenomenon is not surprising. The elevated frequency of C > A mutations could also explain why almost all fish species exhibit transition-to-transversion (Ts:Tv) ratios at the lower end of the vertebrate spectrum (Zhang et al. 2023).

The genome-wide average DNM rates are known to be influenced both by genetic and environmental factors (Houle 1992; Drake et al. 1998). Our estimates were derived from wild-caught parents and their captive born offspring. Given that the meioses during which the detected DNMs occurred likely happened before the parents were brought to captivity, the estimated *µ* should be reflective of the natural DNM rate. To what degree the estimated rate is influenced by environmental vs. genetic factors is not possible to deduce from our data, but whatever the relative importance of these proximate determinants is, the observed rate should be representative of the natural rate in the wild. However, whether the *µ* from this particular Baltic Sea population is representative of that from other three-spined stickleback populations from their Holarctic range remains to be verified. However, given the fairly narrow range of DNM rates in fishes (see Fig. 1 in Zhang et al. 2023), it seems more likely than not that the obtained *µ* estimate is indeed representative for the species. This conjecture is also supported by the fact that the three- and nine-spined stickleback mutation rates were very similar.

Shared DNMs among full-sibs are known to occur before primordial germ cell (PGC) specification or at the early stage of post-PGC (Rahbari et al. 2016; Tang et al. 2016; Jonsson et al. 2021). The proportion of shared mutations among the three-spined stickleback full-sibs was higher than that observed in mice and other fish species (Lindsay et al. 2019; Bergeron et al. 2023), but still lower than that in nine-spined sticklebacks. This could be explained by a higher number of PGCs generated in fish compared to most mammals (Lubzens et al. 2010) increasing the likelihood of early-stage mutations carried by multiple offspring (as illustrated in Supplementary Fig. 2).

Species with higher percentages of shared mutations tend to have lower alpha values, indicating reduced paternal bias in DNM inheritance. Indeed, our results from three-spined sticklebacks provide further evidence for low male bias in fish, similar to findings from the nine-spined stickleback (Zhang et al. 2023) and other fish species (Bergeron et al. 2023). Additionally, we did not observe any sex bias in either the shared mutations occurring post-zygotically or the DNMs arising in the later stages. In vertebrates, no sex bias is expected during the postzygotic period before sexual differentiation of the embryos because germ cells undergo a similar number of replication events to generate spermatogonia and oogonia (Rahbari et al. 2016; Bergeron et al. 2021; 2023). However, a sex difference is expected in mammals as males typically experience more cell replication events in meiosis than females (Rahbari et al. 2016). Unlike most mammals, in which females have a limited number of genome replications of oocytes and males generate much more DNMs after puberty (Rahbari et al. 2016), female fish can produce hundreds to millions of eggs simultaneously from multiple PGCs (as detailed in Supplementary Fig. 2). In addition, fish are seasonal breeders, with males producing sperm only during specific periods of the year (Bergeron et al. 2023), which further reduces the disparity in the number of reproductive cells generated by different sexes. However, lack of sex bias in *µ* could also be due to the narrow range of parental ages in our dataset albeit this hypothesis remains to be tested with known age parents. However, shared DNMs, especially those within repetitive regions, are prone to higher false positive rates compared to the rest of the genome (Burda and Konczal 2023). Although we have verified through sanger sequencing that several shared DNMs and DNMs in repeat regions were true in a study of nine-spined sticklebacks (Zhang et al. 2023, unpublished data), indicating that at least some of the DNMs identified within these regions are likely true, albeit we cannot ascertain that the ones identified in this study are all true. Using PCR or long-read data might help ensure the accuracy of shared DNMs and those in genome regions of low complexity. Furthermore, long-read data would allow future investigations into the mutation rates of indels and structural variants in three-spined sticklebacks.

In conclusion, we have provided the first mutation rate estimate for the three-spined stickleback and found that it is very similar to that of its close relative, the nine-spined stickleback. This is somewhat surprising given that the effective population size of the three-spined stickleback is likely to be higher than that of the nine-spined stickleback (e.g. Merilä 2013; Fang et al. 2020a, 2021; Kemppainen et al. 2021), and because the drift-barrier hypothesis posits that species with smaller effective population sizes are expected to evolve higher mutation rates than those with larger effective sizes (Lynch 2010). Hence, one might have expected to see a lower mutation rate estimate in the three- than in the nine-spined stickleback, but this was clearly not the case. However, given the well documented differences in genomic constitutions and demographic histories across various three-spined stickleback populations (e.g. Fang et al. 2020a), future studies comparing mutation rates of freshwater and marine populations from both Pacific and Atlantic Ocean sites might be of interest.

## Supplementary information

Supplementary Materials

Supplementary data

## Data Availability

The linkage maps applied to calculate the localised recombination rates were from Kivikoski et al. (2022, https://github.com/mikkokivikoski/InverseMappingFunctions). The raw reads of the four three-spined stickleback pedigrees used in this project will be available on ENA (accession ID: PRJEB82216), once the paper is accepted. All the lifted-over annotation files and the programming codes will be accessible on figshare at 10.6084/m9.figshare.27645810 and GitHub at https://github.com/zcharlene/3sticklebackDNMrate.
