# Supplementary material for: Rate of de novo mutations in the three-spined stickleback
Source: Heredity (Edinb). 2025 Jun 12;134(7):387–95. doi: 10.1038/s41437-025-00767-9 (PMC12218844; doi:10.1038/s41437-025-00767-9)
Supplement: Supplementary file 1 — Supplementary Materials [file 41437_2025_767_MOESM1_ESM.pdf]

Supplementary Figure 1. Comparisons of standardised number of DNMs inherited from the two parents on CpG sites (with light grey border) and non-CpG sites (with dark grey border).

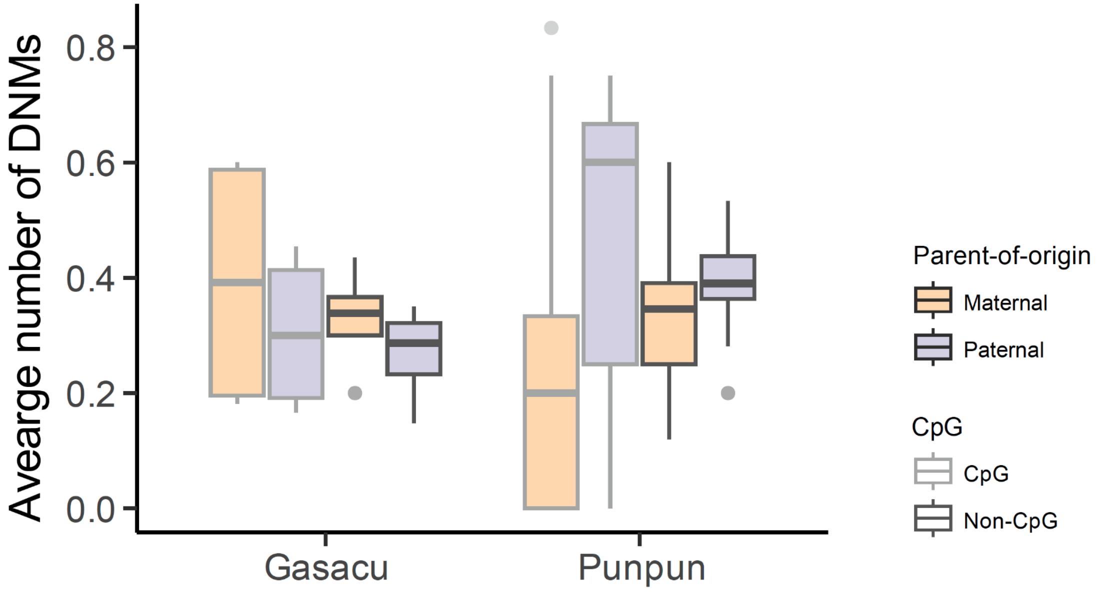

Supplementary Figure 2. A detailed illustration of embryogenesis and gametogenesis in sticklebacks. Spermatogenesis on the left (purple), oogenesis on the right (orange), and the corresponding life stages in the middle (blue). Maternal inheritance of 'nuage' determines the functions of cells after the 1000-cell stage in fish, including germ cells that migrate to the gonads before maturation (represented as yellow cells in the middle life-stage diagram). This process is known as primordial germ cell specification (PGCS). If mutations arise before PGCS or early in the post-PGCS stage (as depicted on the right side of the maternal germline), these mutations will be present in following-stage germ cells and can be shared among siblings. Conversely, if the mutation occurs later during meiosis (as shown on the left side of the paternal germline), the likelihood of this DNM being shared among siblings is little.

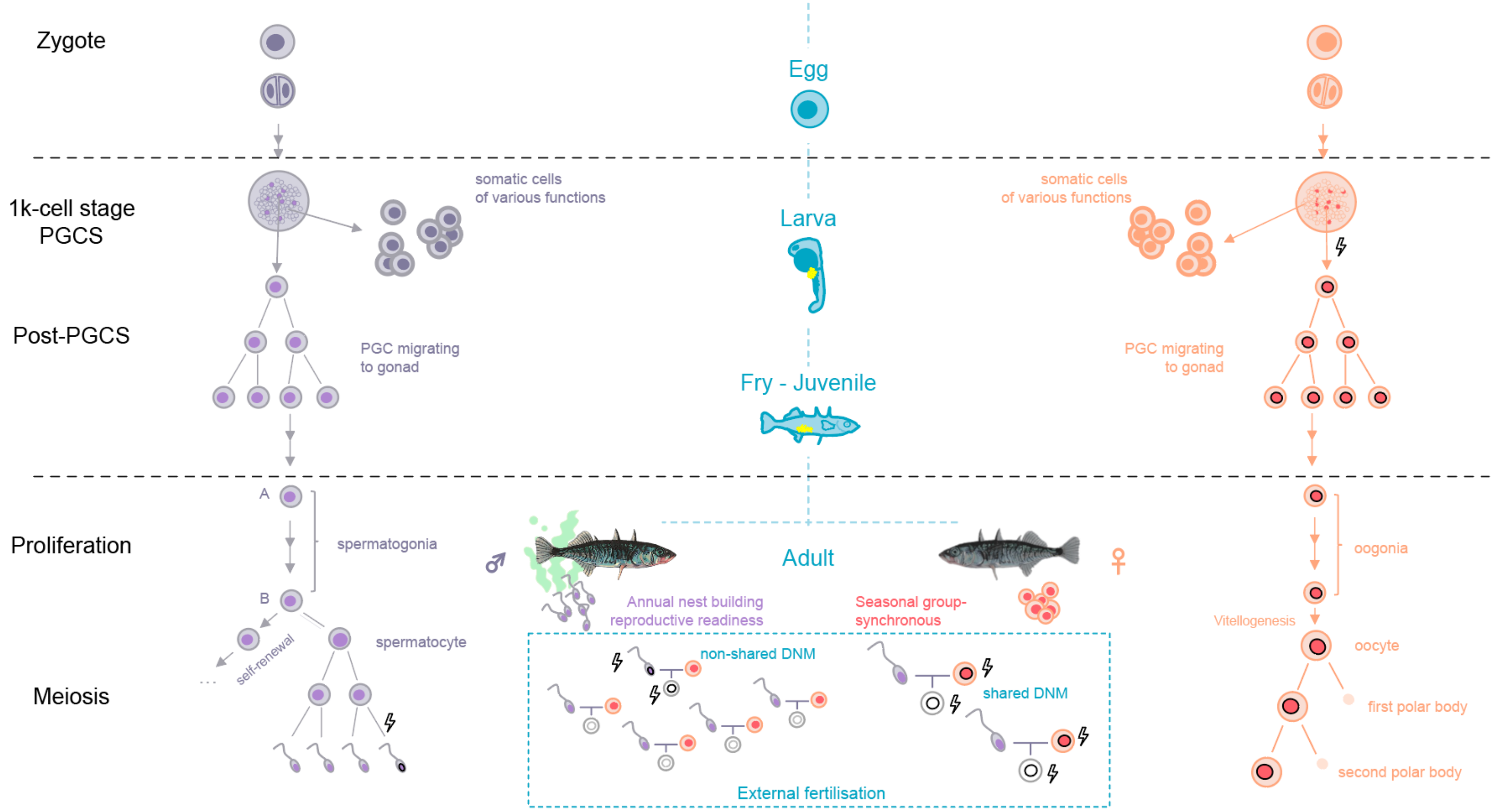

Supplementary Figure 3. (A) A correlation between the per-5Mb CpG contents and the recombination rates. (B) Correlation of the per-chromosomal mutation rates between the two species, accounting for their synteny.

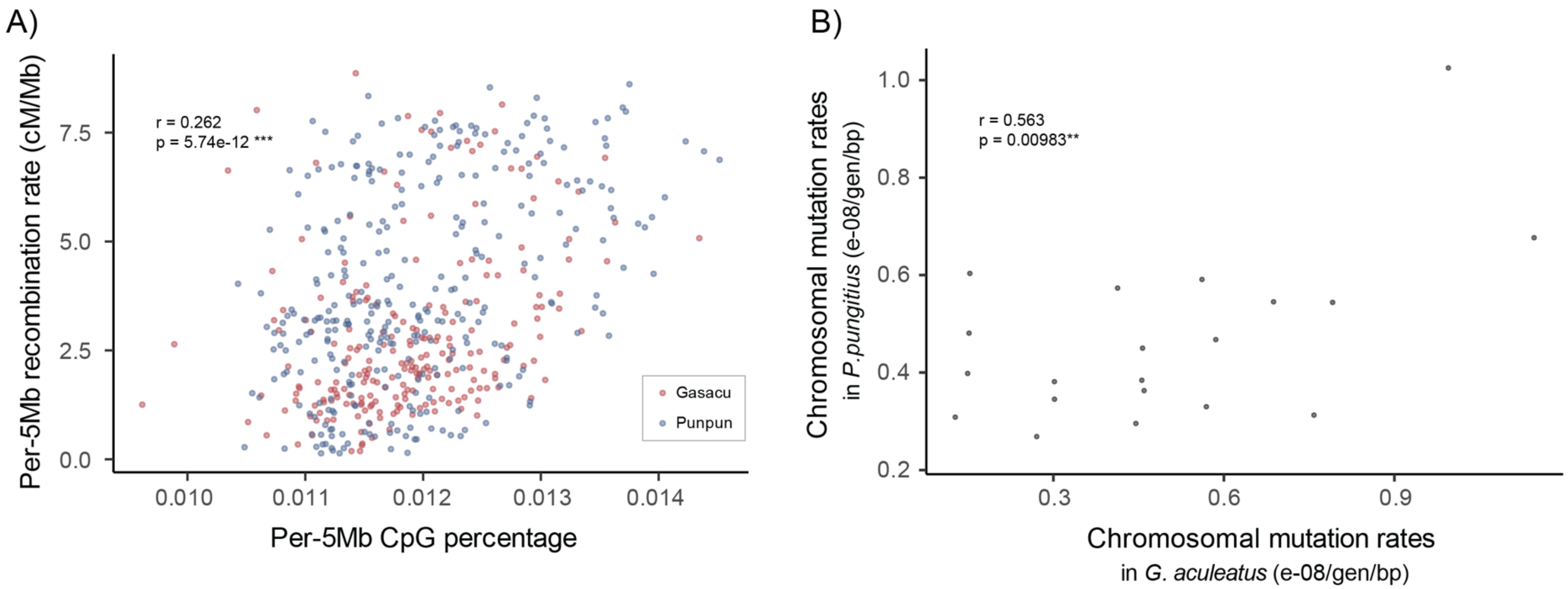

Supplementary Figure 4. A path analysis of the direct and indirect impacts of CpG content and recombination rate on DNM rates.

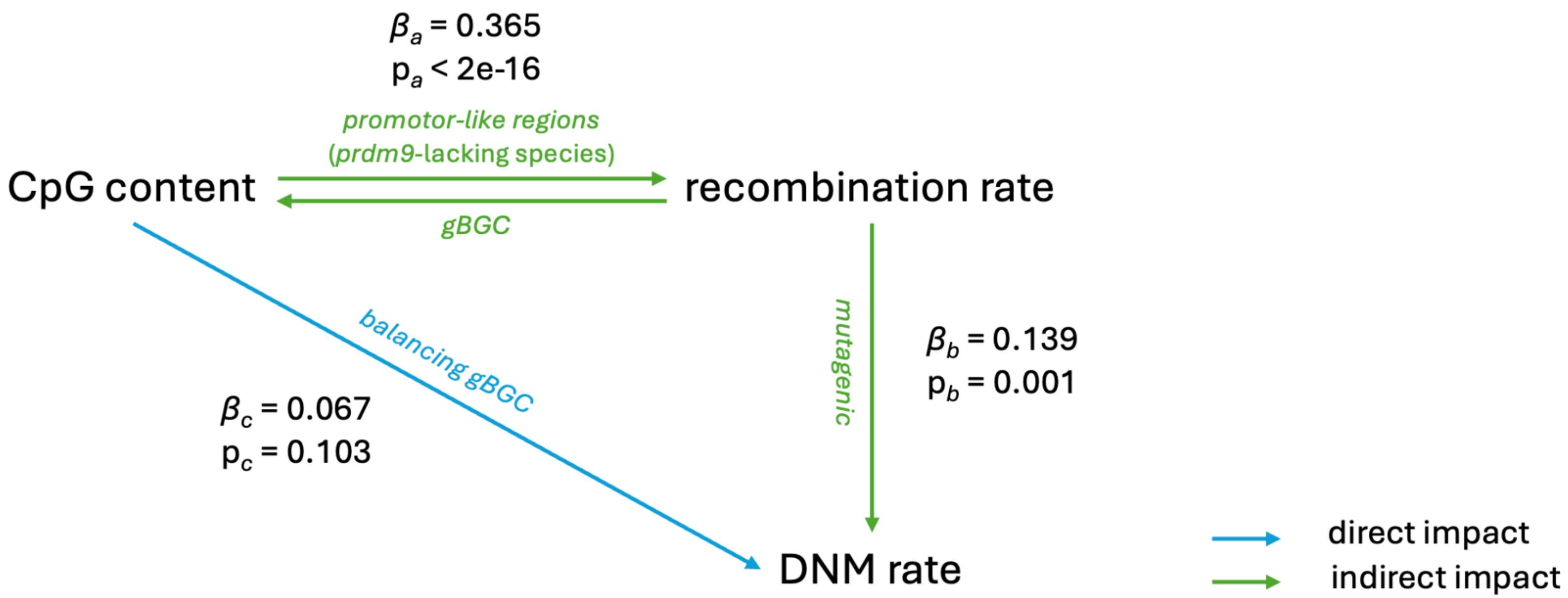

Supplementary Figure 5. Rates of de novo mutations (/bp/generation) compared between A) CG sites located within and outside CpG island, and B) CpG sites and non-CpG sites.

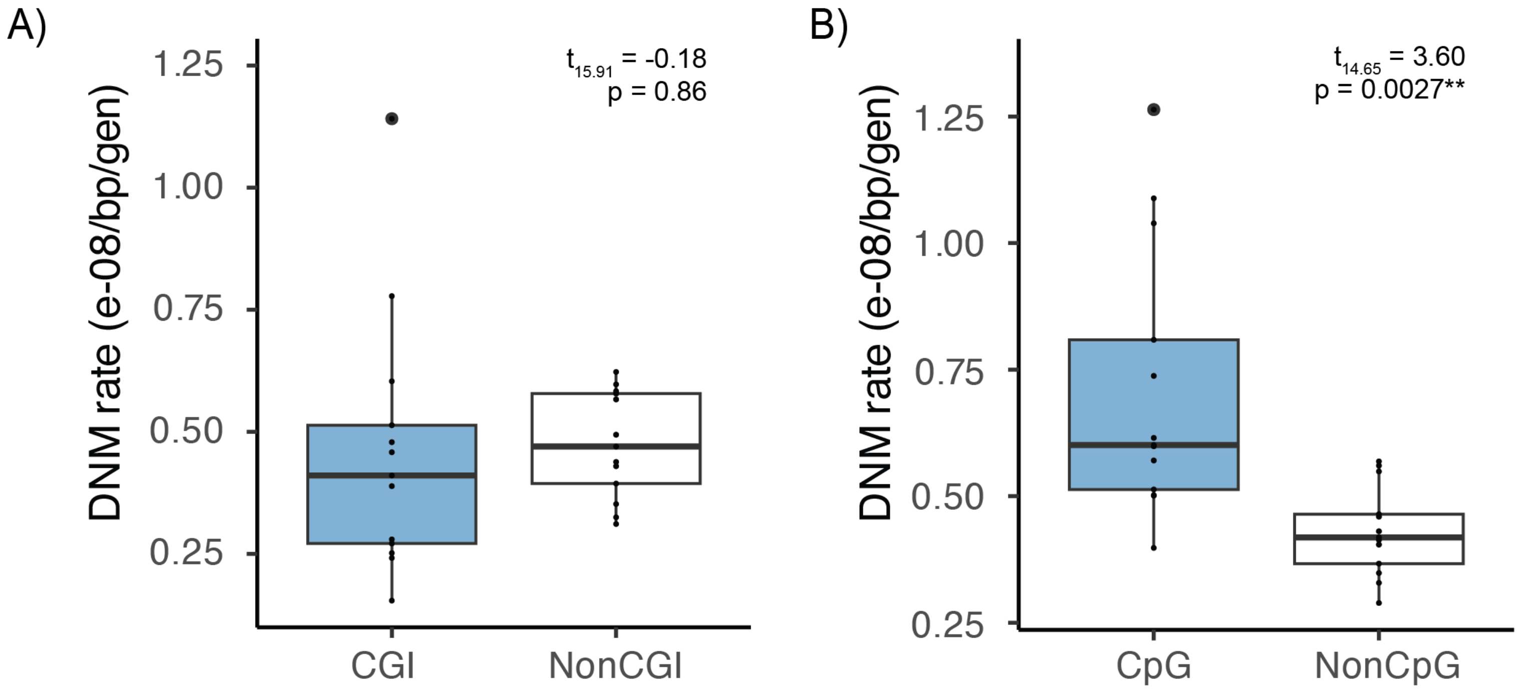

Supplementary Figure 6. An example of true DNM candidate which has passed the IGVtools filter.

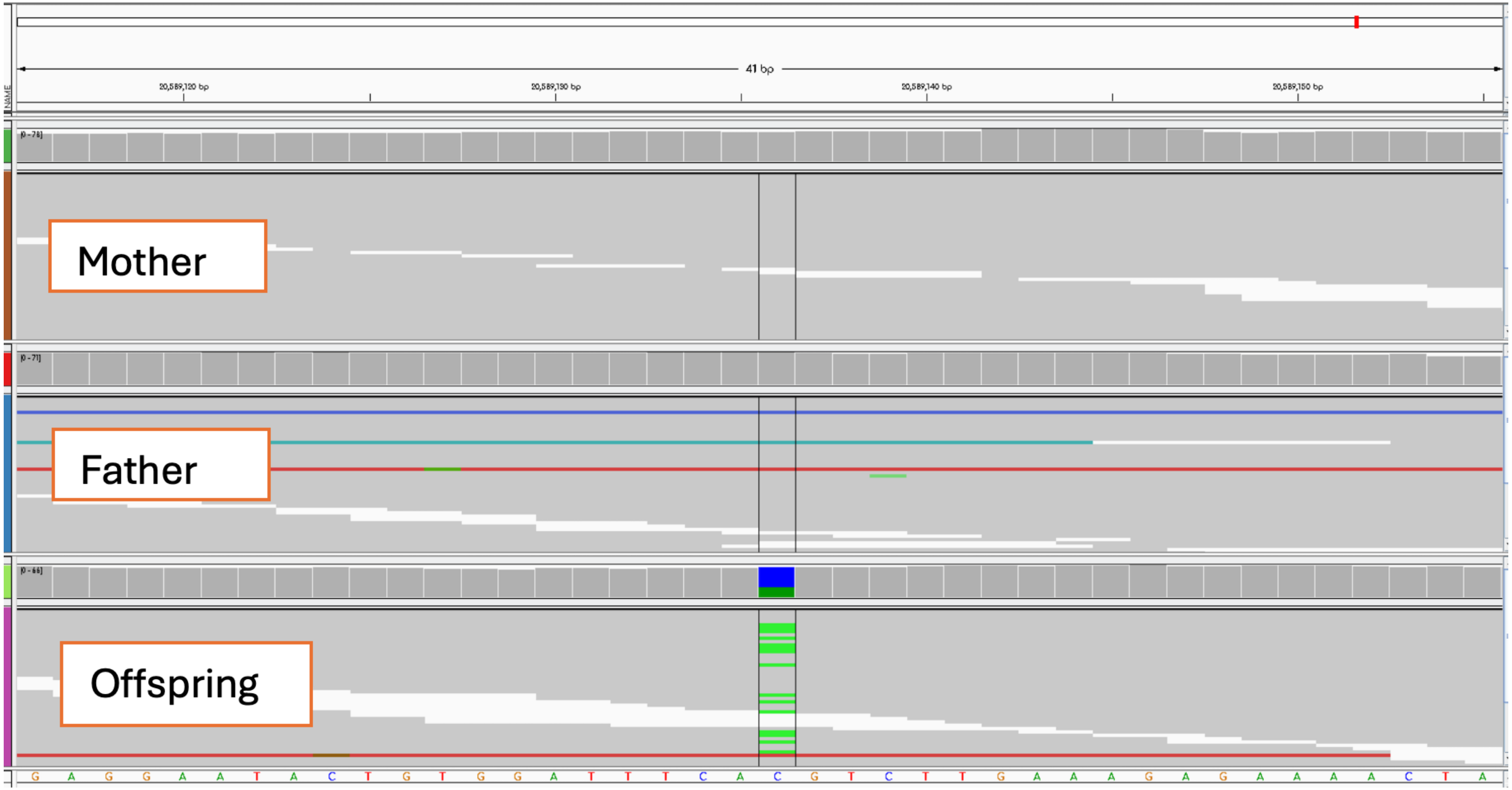

Supplementary Table 1. Filters applied identifying *de novo* mutations (DNM) and estimating the DNM rates.

**Filter**	**Detail**	**Average number of sites removed**	**Average percentage removed**
Site filters	Quality by depth (QD) < 2.0<br>Mapping quality (MQ) < 40.0<br>Fisher's exact test on strand bias (FS) > 60.0<br>Strand odds ratio (SOR) > 3.0<br>Mapping quality rank sum test (MQRankSum) < −12.5<br>Read position rank sum test (ReadPosRankSum) < −8.0	(embedded in the variant calling steps; On average 13152.2 per sample left after mendelian violation)	NA
Individual filters	DNM candidates within 5 bp away from indels<br>genotyping quality (GQ ≤ 80)<br>sequencing depth (DP ≤ 20 and DP ≥ 100)<br>sequencing depth (DP < $0.5DP_{trio}$ and DP > $2DP_{trio}$ for offspring)<br>allelic depth filter (AD1 > 0 for parents)<br>allelic balance (AB < 0.3 and AB > 0.7 for offspring)<br>DNM candidates that occurred in unrelated samples	2360.9<br>9766.5<br>178.6<br><br>1.5<br>810.7<br>23.2	18.0%<br>74.3%<br>1.4%<br><br>0.1%<br>6.2%<br>0.2%
Post-filtering check (False positives)	IGV manual curation and bam-readcount (excluding sites where the parents carry alternative alleles or the offspring do not have a sufficient number of alternative alleles)	27.3	0.2%
Callable genome size	BAM filters (Good Cigar Read Filter, Not Duplicate Read Filter, Passes Vendor Quality Check Read Filter, Mapping Quality Read Filter, Mapping Quality Available Read Filter, Primary Line Read Filter, Mapped Read Filter)<br>Both parents are homozygotes<br>Number of sites passing the trio depth filtering ($0.5DP_{trio} < DP_{child} < 2DP_{trio}$)	NA	NA
False negatives	Number of true heterozygotes deleted by the allelic balance filtering (AB < 0.3 and AB > 0.7)	NA	NA
